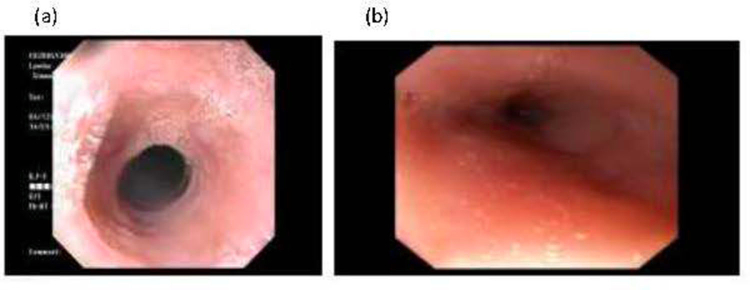# Upadacitinib for Treatment-Refractory Esophageal Lichen Planus: A Retrospective Case Series

**DOI:** 10.1016/j.gastha.2026.100924

**Published:** 2026-03-13

**Authors:** Lina Fikri, Twan Sia, Leeon Bacchus, Aparna Kumar, Ramin Herath, John Leung

**Affiliations:** 1 Boston Specialists, Boston, Massachusetts; 2 Stanford University School of Medicine, Stanford, California

Esophageal lichen planus (ELP) is a rare manifestation of lichen planus (LP), a T-cell-mediated inflammatory condition affecting the skin and mucous membranes.^1^ ELP often presents with dysphagia, heartburn, and epigastric pain. ELP is characterized by friable or tearing mucosa, stenosis, and hyperkeratosis; while on histology, ELP is characterized by lymphocytic infiltration, Civatte bodies, and dyskeratosis. There are currently no consensus guidelines for the treatment of ELP, though swallowed topical corticosteroids and various immunosuppressants may be effective therapies.^2^

Emerging evidence has shown that Janus Kinase (JAK) inhibitors may be effective in treating cutaneous and mucocutaneous LP.^3^^,^^4^ Pro-inflammatory cytokines such as interferon gamma likely play a role in the pathophysiology of LP. JAK inhibitors suppress the signal transduction of these cytokines, thereby treating LP.^3^ However, there currently exists a paucity of data on JAK inhibitors for the treatment of ELP. Isolated case reports have shown that upadacitinib and tofacitinib may improve clinical and histologic features of ELP,^5^^,^^6^ suggesting that JAK inhibitors may also be effective for the treatment of ELP. To our knowledge, there are currently no case series describing the use of JAK inhibitors for the treatment of ELP.

Thus, in this retrospective case series, we investigate the effects of upadacitinib, an oral selective JAK-1 inhibitor, on ELP.

We conducted a chart review of patients with ELP who tried upadacitinib at a single multidisciplinary clinic (gastroenterology and allergy/immunology). The electronic medical record was searched between 2017 and 2025 using the *International Classification of Disease, Tenth Revision* codes L43.9 [LP] and L43.8 [other LP]. Patients (1) without clinical or histologic evidence of ELP and (2) who never started upadacitinib were excluded.

Primary end points for this study were clinical, histologic, and endoscopic changes after starting upadacitinib. During the data abstraction process, 1 investigator collected progress notes at baseline and while on upadacitinib. Then, protected health information, dates, and mentions of treatment were removed using the available National Library of Medicine’s NLM-Scrubber tool.^7^ Next, 3 separate trained investigators, blinded to the study hypothesis, scored symptoms preupadacitinib and on-upadacitinib. Symptoms were scored on a 4-point scale (0 = “absent,” 1 = “mild,” 2 = “moderate,” 3 = “severe”), which were summed and averaged between the 3 investigators to form the average total symptom score (ATSS). Symptoms included in the ATSS for ELP were dysphagia, odynophagia, heartburn, regurgitation, chest pain, hoarseness, chronic cough, weight loss, and oral lesions.^2^ Symptoms were determined a priori based on existing literature describing prominent clinical features of ELP. ATSS for ELP had a minimum symptom score of 0 and a maximum of 27 (most severe symptoms). To ensure interrater reliability, an intraclass correlation constant (ICC) was calculated using a 2-way, mixed effects model with absolute agreement. ICC was 0.94, which was acceptable reliability based on a priori criterion (ICC≥0.70). Histologic and endoscopic data were abstracted and compared preupadacitinib vs on-upadacitinib descriptively.

Three patients with ELP had tried upadacitinib and were included in our study. All 3 patients with ELP were female, with an average age of 66.98 years (range: 57.8–74.4 years). Two patients were diagnosed with oral LP prior to being diagnosed with ELP.

At the time of diagnosis, patients experienced various ELP symptoms: dysphagia (n = 3), odynophagia (n = 2), heartburn (n = 2), chest pain (n = 2), chronic cough (n = 1), weight loss (n = 1), and oral lesions (n = 1). The mean ATSS at baseline was 8.44 (range: 3.33–15). Of note, patient 3 underwent 6 esophageal dilations and had a percutaneous endoscopic gastrostomy tube placed to manage dysphagia.

Before starting upadacitinib, all 3 patients failed swallowed corticosteroids. Patient 1 did not have histologic improvement of ELP on budesonide 3 mg 3 times daily and was diagnosed with iatrogenic Cushing syndrome with low adrenocorticotropic hormone while on long-term treatment with budesonide. Adrenocorticotropic hormone levels returned to normal after discontinuing budesonide. Patient 2 symptomatically failed fluticasone propionate 220 mcg 2 puffs twice daily and reported ecchymoses and skin atrophy. Patient 3 reported significant relief of dysphagia with budesonide 3 mg 3 times daily but developed adrenal insufficiency, osteoporosis, and ecchymoses, deemed by their endocrinologist to be related to long-term treatment with corticosteroids.

In addition, all patients failed high-dose proton pump inhibitor therapy, and most failed systemic immunosuppressive agents (patient 1 failed swallowed tacrolimus 1 mg daily, patient 3 failed mycophenolate mofetil 750 mg twice daily).

Patients then started upadacitinib 15 mg daily. Symptom scores were evaluated after an average of 29.7 weeks following upadacitinib initiation (range: 43.6–21.7 weeks). All patients’ ATSS improved from baseline (mean: ATSS 1.11, range: 0–2).

In addition, all individual symptom scores improved, except for patient 2, who experienced new odynophagia and voice hoarseness (Figure). Endoscopic and histologic improvement of ELP was observed in all patients. Patients underwent repeat endoscopy after an average of 20.67 weeks on upadacitinib (range: 16–26 weeks). Endoscopic features for patients 1 and 3 completely normalized, whereas patient 2 exhibited a persistent 14 mm stricture following treatment with upadacitinib (Table). Notably, patient 3 did not require repeat esophageal dilations after initiating upadacitinib therapy.

**Figure:**
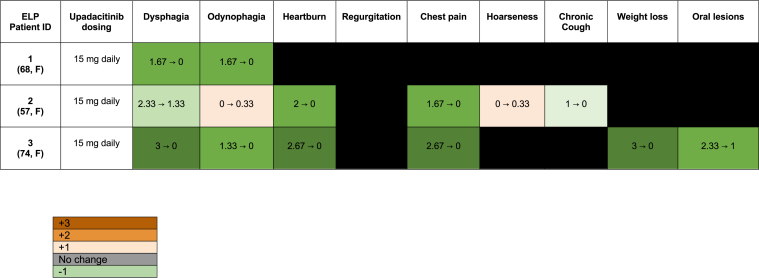
Summary plot of individual clinical changes before and after treatment with upadacitinib in patients with esophageal lichen planus (ELP). Scores were assigned based on the following atlas: 0 = absent, 1 = mild, 2 = moderate, 3 = severe. Symptoms are reported as “diagnostic score → “on upadacitinib score” and were averaged across scores from 3 blinded raters. Orange denotes increase in score (symptom exacerbation), gray denotes no change in score, and green denotes decrease in score (improvement of symptoms). Black indicates that the symptom was absent before and after treatment with upadacitinib.

**Table:** Clinical, Histologic, and Endoscopic Presentations of Patients With Esophageal Lichen Planus (ELP) at Baseline and on Treatment With Upadacitinib

Patient ID (age in years, sex); upadacitinib dosage and duration of treatment at follow-up endoscopy	Previously failed treatments	EGD time point	Histologic end points	Endoscopic end points	Average total symptom score (ATSS) at diagnosis → on-upadacitinib
Patient 1 (68, F); upadacitinib 15 mg once daily for 26 wk	Sucralfate 1 mg daily; tacrolimus 1 mg daily; budesonide 3 mg 3 times daily; pantoprazole 40 mg once daily	Baseline	Marked epithelial lymphocytic infiltration, dyskeratosis, and Civatte bodies	Narrowing of the lumen of the entire esophagus	3.33 → 0.33
		On-upadacitinib	Resolution of lymphocytic infiltration	Endoscopically normal	
Patient 2 (57, F); upadacitinib 15 mg once daily for 20 wk	Famotidine 20 mg twice daily and fluticasone propionate 220 mcg 2 puffs twice daily; omeprazole 40 mg twice daily	Baseline	Increased lymphocyte infiltration in the proximal, mid and distal esophagus	Mild circumferential ridges, mild furrows without visible depth, 14 mm stricture present. White plaques throughout the entire esophagus	7 → 2
		On-upadacitinib	Resolution of lymphocytic infiltration. Squamous epithelium with patchy chronic inflammation	14 mm stricture present	
Patient 3 (74, F); upadacitinib 15 mg once daily for 16 wk	Rabeprazole 20 mg daily; budesonide 3 mg twice daily; azathioprine 50 mg daily and prednisone 7.5 mg daily; mycophenolate mofetil 500 mg twice daily; mycophenolate mofetil 750 mg twice daily and PPI 20 mg daily; vedolizumab 300 mg every 8 wk	Baseline	Severe ulcerative esophagitis. Increased intraepithelial lymphocytes in the mid and distal esophagus	Esophagitis with non-bleeding superficial esophageal ulcer at 20 cm. Benign-appearing esophageal stenoses with luminal narrowing and mucosal sloughing	15 → 1
		On-upadacitinib	Resolution of lymphocytic infiltration	Endoscopically normal	

Baseline is defined as the esophagogastroduodenoscopy (EGD) where the patient was diagnosed with ELP. At the baseline EGD, patient 1 was on budesonide 3 mg three times daily, patient 2 was on dupilumab 300 mg weekly (for eosinophilic esophagitis), and patient 3 was on mycophenolate mofetil 750 mg twice daily and proton pump inhibitor therapy 20 mg once daily.

One patient (patient 3) experienced a suspected transient ischemic attack while on upadacitinib, but the exact cause could not be determined. With agreement from the patient’s neurologist, upadacitinib was prophylactically reduced to 7.5 mg daily. Patient 3 maintained symptomatic relief on upadacitinib 7.5 mg daily, although histologic findings worsened, showing mild esophagitis and ridges. No other adverse events could be attributed to upadacitinib.

ELP is a difficult-to-treat lymphocyte-mediated inflammatory condition without established treatment guidelines.^1^^,^^2^ In this retrospective case series, upadacitinib, a selective JAK-1 inhibitor, showed clinical, histologic, and endoscopic benefits in all ELP patients, providing preliminary evidence for its potential efficacy and tolerability for ELP treatment.

All patients in our study failed standard therapies including swallowed corticosteroids, high-dose proton pump inhibitors, and systemic immunosuppressants, underscoring the need for alternative therapies, especially in patients with severe disease requiring repeated esophageal dilations.

Limitations of our study include our single center, small sample size, and retrospective design. Future well-designed, larger prospective trials are needed to confirm our findings.

## Figures and Tables

**Supplementary Figure 1:**